# Effects of the replacement of fishmeal by soy protein concentrate on growth performance, apparent digestibility, and retention of protein and amino acid in juvenile pearl gentian grouper

**DOI:** 10.1371/journal.pone.0222780

**Published:** 2019-12-23

**Authors:** Yan Chen, Jun Ma, Hai Huang, Honggan Zhong

**Affiliations:** 1 Hainan Key Laboratory for Conservation and Utilization of Tropical Marine Fishery Resources, Hainan, PR China; 2 College of Life Science and Ecology, Hainan Tropical Ocean University, Sanya, PR China; 3 Sanya Municipal Bureau of Ocean and Fishery, Hainan, PR China; University of Illinois, UNITED STATES

## Abstract

Soy protein concentrate (SPC), as a protein source, is widely used to replace partial fishmeal (FM) in aquafeeds, especially for carnivorous fish. This study investigated the effects of partial FM replacement by SPC for juvenile pearl gentian grouper. The fish were fed with diets containing six levels of SPC (SPC 0, 15, 30, 45, 60, and 75) for 6 weeks. At the end of the feeding trial, average body weight gain (BWG), specific growth ratio (SGR), and weight gain ratio (WGR) had the highest values in fish fed with diet SPC 15, followed by that of fish fed with SPC 0 while fish fed with SPC 75 had the lowest values (*P* < 0.05). Fish fed with diet SPC 15 and SPC 30 had the highest protein efficiency ratio (PER) while fish fed with diet SPC 15, SPC 30, and SPC 45 had the highest feed conversion ratio (FCR) (*P* < 0.05). Daily feed intake (DFI) was significantly decreased in fish fed with diets containing any level of SPC (*P* < 0.05). Survival rate was significantly higher in fish fed with diets SPC 15, SPC 30, and SPC 45 as compared to other treatments. Fish fed the diet including less than 30% FM replacement showed a higher protein content in the muscle. The ADC of dietary protein and some amino acids were significantly higher in diets SPC 0, followed by SPC 15; while SPC 75 had the lowest content (*P* < 0.05). Similarly, fish fed with SPC 30 and SPC 15 showed significantly higher protein and amino acid (AA) retention than other dietary treatments. The optimal FM replacement with SPC for specific growth ratio (SGR) was estimated to be 14.41% using a non-linear higher order regression model. These results indicated that pearl gentian grouper has a limited ability to utilize SPC as a protein source, and the FM replacement with SPC should be less than 30% (FM45.5 g 100g ^-1^ and SPC18g 100g ^-1^).

## Introduction

The aquaculture industry is rapidly expanding with an annual growth rate of over 7%. Thus, the demand for commercial aquafeeds is steadily growing to meet this expansion [1, 2]. This has also led to an increased requirement for fishmeal (FM) that is a major protein source in commercial aquafeeds and is obtained from marine forage fish species [3, 4]. It is predicted that FM production will decline in the future due to the reduction in capture fishery resources, and as a result the price of FM is constantly increasing [5, 6]. It has long been recognized that the reliance on fishmeal is a risk for the aquaculture industry [7]. Therefore, it is essential to find alternative protein sources for the sustainable development of the aquaculture industry.

Soybean meal is one of the most suitable alternatives to FM for aquatic animal feed due to its high protein content, good balance of essential amino acids (EAA), and cheaper cost [8, 9] However, the presence of some anti-nutritional factors (ANFs) in soybean meal may result in adverse effects on digestion or absorption of nutrients for some species [10, 11]. Thus, other soy protein sources excluding or containing a small amount of ANFs were found and used in replacement of FM [12, 13]. Soy protein concentrate (SPC), made by moving a portion of the carbohydrates (sugars) from dehulled and defatted soy flakes through aqueous ethanol, has similar content of crude proteins and EAA as compared to FM along with with lower ANFs [14, 15]. Many studies have assessed the influence of SPC on fish during the past 20 years, and the results showed that different species had unequal levels of tolerance towards SPC. Several studies have demonstrated that SPC can effectively replace FM as a protein source in the diet of *Salmo salar* [16], *Oncorhynchus mykiss* [17], and *Trachinotus ovatus* [18]. But some fish could condone less than 40% FM to be substituted by SPC in the feed of juvenile starry flounder (*Platichthys stellatus*), carp (*Cyprinus carpio*) and yellowtail (*Seriola quinqueradiata*) [19–21].

Pearl gentian grouper, a hybrid species (*Epinephelus lanceolatus ♂ × E*. *fuscoguttatus ♀*), is an important commercial and economic fish which grows rapidly, has strong disease resistance and is highly nutritious [22]. This species has been widely cultured in China using land-based and sea-cage farming techniques, and was mainly fed with formulated pellet diets [23]. As a typical carnivorous species, pearl gentian grouper requires high protein, and is heavily dependent on high levels of FM in the diet to meet its protein requirement, which leads to higher production costs. Previous reports have listed the nutrient requirements of protein [24], lipid [25], fatty acid for grouper [26]. But few studies have focused on the effects of substituting FM with SPC in grouper feed [27]. Also, it is still unknown whether it is possible to replace FM with SPC for pearl gentian grouper. Thus, SPC was used as the source of protein to replace FM in the present study, and the growth performance, apparent digestibility, and retention of protein and amino acid were observed.

## Materials and methods

### Diet formulation

FM and SPC were purchased from Rifeng animal husbandry Co. LTD. (Guangzhou, China). Other feed ingredients were obtained from Xinnong Feed Company (Shanghai, China). Six isonitrogenous and isocaloric diets (46% crude protein, 18 MJ/kg gross energy) were formulated following the method reported by Shiau & Lan [24] and Luo et al [25]. Among these diets, SPC replaced 0%–75% of FM protein (SPC0, SPC15, SPC30, SPC45, SPC60, and SPC75). Chromium oxide (Cr_2_O_3_, Guangzhou Green Bank Trade Co., LTD) was appended as a dietary inert marker for the determination of digestibility (Table 1).

**Table 1:** Ingredients and proximate compositions of the experimental diets (as dry-matter basis %).

Ingredients (g 100g^−1^)	Diets					
	SPC0	SPC15	SPC 30	SPC45	SPC60	SPC75
Fishmeal 60%^(1)^	65.0	55.3	45.5	35.8	26.0	16.3
Soy protein concentrate 65%^(2)^	0.0	9.0	18.0	27.0	36.0	45.0
Casein	5.0	5.0	5.0	5.0	5.0	5.0
Shrimp meal	2.5	2.5	2.5	2.5	2.5	2.5
Wheat flour	18.0	17.8	17.5	17.3	17.0	16.8
Binding agents	2.0	2.0	2.0	2.0	2.0	2.0
Soybean oil	1.0	1.5	2.0	2.5	3.0	3.5
Fish oil	1.0	1.5	2.0	2.5	3.0	3.5
Squid visceral ointment	1.5	1.5	1.5	1.5	1.5	1.5
Vitamin premix^(3)^	1.0	1.0	1.00	1.0	1.0	1.0
Mineral premix^(4)^	1.0	1.0	1.0	1.0	1.0	1.0
Choline chloride	0.5	0.5	0.5	0.5	0.5	0.5
monocalcium phosphate	1.0	1.0	1.0	1.0	1.0	1.0
Cr_2_O_3_	0.5	0.5	0.5	0.5	0.5	0.5
Proximate composition						
Crude protein	46.56	46.49	47.11	46.12	46.85	46.3
Crude lipid	9.63	9.37	9.29	9.10	9.00	9.15
Crude ash	15.73	14.56	13.34	11.85	10.49	9.26
ANFs (μg /100g) ^(5)^	0	0.68× 10^5^	1.43 × 10^5^	2.25× 10^5^	2.96 × 10^5^	3.50× 10^5^
Calculated gross energy (kJ g^−1^)	0.079	0.078	0.079	0.079	0.081	0.081

^(1)^ Fish meal: crude protein 60%, crude lipid 6%, ash 21% (dw), Lys 5.72%, Arg 4.31%, His 2.16%, Asp 6.28%, Glu 9.05%, Gly 4.18%, Ala 4.29%, val 2.41%, Leu 5.21%, Ile 2.40%, Phe 2.95%, Pro 2.37%, Trp 0.78%, Tyr 1.06%, Ser 2.66%, Met 2.23%, Thr 3.09%.

^(2)^ Soy protein concentrate: crude protein 65.0%, crude lipid 1.0%, crude fiber 4.00%, ash 4.8%, soluble nitrogen-free extract 2.2% and insoluble nitrogen-free extract about 15% (dw), phytic acid 5.1 × 10 ^5^ μg/100g, raffinos 1.1 × 10^5^μg /100g, glycinin 9.04×10^4^μg/100g, β-conglycinin 0 μg/100g, trypsin inhibitor 2.1×10^4^μg/100g; Oligosaccharides, stachyose, lectins, sponins and urease activity cannot detected; Lys 4.06%, Arg 4.81%, His 1.54%, Asp 7.07%, Glu 13.55%, Gly 3.08%, Ala 3.11%, val 3.38%,Leu 5.24%, Ile 3.12%, Phe 3.23%, Pro 3.86%, Trp 0.97%, Tyr 2.35%, Ser 3.55%, Met 0.99%,Thr 2.62%.

^(3)^ Vitamin mixture (mg kg^−1^ diet): retinol acetate, 38.0; cholecalciferol, 13.2; a-tocopherol, 210.0; thiamin, 115.0; riboflavin, 380.0 pyridoxine 88.0; pantothenic acid, 368.0; niacin acid, 1030.0 biotin, 10.0; folic acid, 20.0; vitamin B12, 1.3; inositol, 4000.0; ascorbic acid, 500.0 (Ding et al. 2010).

^(4)^ Mineral mixture (mg kg^−1^ diet): MgSO_4_·7H_2_O, 3568.0; NaH_2_PO_4_·2H_2_O, 25568.0; KCl, 3020.5; KAl (SO_4_)_2_, 8.3; CoCl_2_, 28.0; ZnSO_4_·7H_2_O, 353.0; Ca-lactate, 15968.0; CuSO_4_·5H_2_O, 9.0; KI, 7.0; MnSO_4_·4H_2_O, 63.1; Na_2_SeO_3_, 1.5; C_6_H_5_O_7_Fe·5H_2_O, 1533.0; NaCl, 100.0; NaF, 4.0 (Ding et al. 2010).

^(5)^ ANFs of diets: SPC0: 0; SPC 15: phytic acid 4.57 × 10^4^ μg/100g, raffinos 0.99× 10^4^ μg /100g, glycinin 1.1× 10^4^ μg/100g, β-conglycinin 0 μg/100g, trypsin inhibitor 1100μg/100g; SPC30: phytic acid 0.90× 10^5^ μg/100g, raffinose 0.20× 10^5^ μg/100g, glycinin 3.02 × 10^4^ μg/100g, β-conglycinin 0 μg/100g, trypsin inhibitor 3.1× 10^3^ μg/100g; SPC45: phytic acid 1.36 × 10^5^ μg/100g, raffinose 0.30 × 10^5^μg/100g, glycinin 5.04 × 10^4^ μg/100g, β-conglycinin 0 μg/100g, trypsin inhibitor 8.2 × 10^3^ μg/100g; SPC60: phytic acid 1.82× 10^5^μg/100g, raffinose 0.39× 10^5^μg/100g, glycinin 6.03× 10^4^ μg/100g, β-conglycinin 0 μg/100g, trypsin inhibitor 1.4 × 10^4^μg/100g;SPC75: phytic acid 2.12 × 10^5^μg/100g, raffinose 0.50 × 10^5^μg/100g, glycinin 7.04 × 10^4^ μg/100g, β-conglycinin 0 μg/100g, trypsin inhibitor 1.4× 10^4^μg/100g. Oligosaccharides, stachyose, lectins, sponins and urease activity cannot be detect.

After shredding and passing through a 250 μm sieve, all the ingredients in each diet were weighed and mixed with fish oil and distilled water (30%, v/w) and made into a stiff dough. The dough was then formed into 3-mm diameter pellets using the twin-screw extruder (F-26 (II), South China University of Technology, China), and dried at 60°C in an oven. All diets were stored at -20°C until used. Table 1 shows the proximate composition and ANF_S_ of the diets and Table 2 shows the amino acid composition of the diets.

**Table 2:** Amino acid composition of the diets (g kg^−1^ dry weight for free amino acid and % for hydrolytic amino acids).

Items	Diets											
	SPC0		SPC15		SPC 30		SPC45		SPC60		SPC75	
	hydrolytic	free	hydrolytic	free	hydrolytic	free	hydrolytic	free	hydrolytic	free	hydrolytic	free
Polar basic amino acids												
Lysine	2.41	0.75	2.24	0.64	2.31	0.73	2.20	0.63	2.12	0.48	1.98	0.36
Arginine	2.26	0.56	2.26	0.56	2.44	0.74	2.47	0.74	2.67	0.73	2.59	0.69
Histidine	0.62	0.66	0.68	0.64	0.70	0.50	0.72	0.45	0.78	0.37	0.79	0.25
Polar acidic amino acids												
Aspartic acid	3.89	0.48	3.96	0.43	4.23	0.39	4.37	0.34	4.54	0.29	4.56	0.21
Glutamic acid	6.43	0.93	6.55	0.79	6.99	0.75	7.29	0.62	7.71	0.47	7.76	0.36
Non-polar amino acids												
Isoleucine	1.11	0.68	1.03	0.54	1.10	0.57	1.12	0.47	1.05	0.36	0.85	0.24
Leucine	3.10	1.32	2.93	1.06	3.09	1.17	3.09	0.97	3.12	0.73	2.91	0.50
Valine	1.26	0.93	1.23	0.76	1.23	0.79	1.27	0.65	1.15	0.49	1.13	0.32
Glycine	2.82	0.65	2.59	0.44	2.48	0.42	2.37	0.34	2.29	0.28	2.00	0.21
Alanine	2.90	1.87	2.68	1.55	2.62	1.49	2.51	1.21	2.47	0.90	2.18	0.63
Phenylalanine	1.49	0.49	1.52	0.40	1.63	0.41	1.66	0.35	1.75	0.28	1.73	0.19
Polar neutral amino acids												
Tryptophan	na	na	na	na	na	na	na	na	na	na	na	na
Methionine	0.85	0.27	0.63	0.25	0.74	0.20	0.73	0.19	0.61	0.07	0.49	0.05
Serine	1.81	0.25	1.85	0.22	1.98	0.20	2.12	0.17	2.19	0.14	2.21	0.10
Threonine	1.55	0.33	1.62	0.28	1.52	0.28	1.47	0.23	1.51	0.18	1.49	0.12
Tyrosine	0.55	0.29	0.62	0.24	0.73	0.23	0.74	0.20	0.78	0.15	0.73	0.11
Taurine	0.23	1.68	0.19	1.31	0.18	1.24	0.15	0.98	0.09	0.67	0.07	0.43
Cysteine	0.26	0.35	0.25	0.42	0.30	0.45	0.32	0.60	0.35	0.95	0.31	1.20
ΣEAA^(1)^	14.65	5.97	14.17	5.12	14.75	5.40	14.72	4.68	14.76	3.68	13.95	2.72
ΣNEAA^(2)^	18.80	6.50	18.90	5.40	19.50	5.15	19.78	4.45	20.41	3.85	19.80	3.06
EAA/NEAA	0.8	0.9	0.8	1.0	0.8	1.1	0.7	1.1	0.7	1.0	0.7	0.9

^(1)^ ΣEAA: sum of essential amino acids

^(2)^ ΣNEAA: sum of non-essential amino acids

### Feeding trial

Prior to the experiment, the use of animals was approved by the "Institutional Animal Care and Use Committee of Hainan Tropical Ocean University" and "Hainan Key Laboratory for Conservation and Utilization of Tropical Marine Fishery Resources" (20161111A1). Pearl gentian grouper was purchased from a commercial hatchery (Hainan, China). Before the trial, fish were stocked in an indoor recirculating aquaculture system (Hainan Key Laboratory for Conservation and Utilization of Tropical Marine Fishery Resources, College of life science and ecology, Hainan Tropical Ocean University, Sanya, China) for 2 weeks to adapt to the feeding environment. The fish were fed with a commercial diet (Nisshin Flour Milling Co., Ltd., Japan) twice daily for satiation.

The fish (8.00 g ± 0.10) were randomly distributed in 18 tanks of a 10 m^3^ indoor sea water circulating system (500 L per tanks, 50 fish per tank) and each diet had triplicate tanks. The fish were fed twice a day (08:00 am and 16:00 pm). Throughout the trial, feed intake of each diet and mortality of the fish were recorded in each tank. After feeding the fish with diet in each tank for 30 min, the uneaten diet was siphoned out and dried overnight at 50°C before being weighed to avoid any contamination with feces. And the weight of the uneaten diet was subtracted to calculate the daily feed intake in each tank.

The feces were collected after removing the uneaten feed pellets per diet at 8:30pm-9:00pm daily for 30 days (from day 12 to day 42 of the feeding trial). The feces were collected from the tank floor by siphoning and were surrounded with ice, and extra attention was intended to this procedure in order to avoid contamination or misleading samples. Feces were subsided and kept at −20°C until analyzed.

Water quality was monitored daily was found consistent for the following parameters: temperature (29.2 ± 0.4°C), dissolved oxygen (7.10 ± 0.2 mg L^−1^), salinity (25.8 ± 0.5‰), pH (7.2 ± 0.2), and total ammonia nitrogen (0.3 ± 0.2 mg L^−1^). This study followed good laboratory practices (GLP).

### Sample collection and calculation formula for growth performance

The fish were euthanized by rapid cooling and tricaine methanesulfonate (MS222), based on the method of Wilson et al. [28]. At the beginning of the trial, 20 fish were euthanized, ground into homogeneous slurry, freeze-dried, reground, and immediately frozen at -20°C for detecting the whole-body composition. After six weeks, all the fish were harvested, anaesthetized using MS-222 (50 mg L^−1^, 3-aminobenzoic ethyl ester acid, Sigma, USA), and weighed. Specific growth rate (SGR), survival, feed conversion ratio (FCR), and protein efficiency ratio (PER) were calculated. At the end of the trial, ten fish from each tank were euthanized, and immediately frozen at -20°C for determination of the whole-body composition.

Eight fish from each tank were anaesthetized, weighted, and dissected to remove stomach, liver, and intestines. The weight of the liver and intraperitoneal fat was recorded to calculate the hepatosomatic index (HSI) and the intraperitoneal ratio (IPR). The dorsal muscle was collected for the proximate composition analysis. Feces were collected after removal of the extra and uneaten feed pellets per meal for 15 days (from day 27 to day 42 of the feeding trial) and kept at −20°C for analysis.

The computational formulas were as follows:

$$\mathrm{Average}\;\mathrm{body}\;\mathrm{weight}\;\mathrm{gain}\;(\mathrm{BWG},\;g)=\left(W_{f}{‐W}_{i}\right)/\mathrm{amount}\;\mathrm{of}\;\mathrm{fish};$$

$${\mathrm{SGR}\;(\%\;d}^{-1})=100\times \left({\mathrm{In}\;W}_{f}{‐W}_{i}\right)/t;$$

$$\mathrm{FCR}=F/\left(W_{f}{‐W}_{i}\right);$$

Survivalrate(SR,%)=100×(finalamountoffish)/(initialamountoffish);

$$\mathrm{Weight}\;\mathrm{gain}\;\mathrm{ratio}\;\left(\mathrm{WGR}\right)=\left(W_{f}{‐W}_{i}\right)/W_{i};$$

$$\mathrm{Daily}\;\mathrm{feed}\;\mathrm{intake}\;\left({\mathrm{DFI},\;\%\;d}^{-1}\right)=100\times \mathrm{dry}\;\mathrm{feed}\;\mathrm{intake}/\left(\left(\mathrm{initial}\;\mathrm{feed}\;\mathrm{weight}+\mathrm{final}\;\mathrm{weight}\right)/2\times t\right);$$

PER=wetweightgain/proteinintake;

$$\mathrm{Condition}\;\mathrm{factor}\;\left({\mathrm{CF},\;g\;\mathrm{cm}}^{-3}\right)=100\times \left(W_{f}/L^{3}\right);$$

$$\mathrm{HSI}\left(\%\right)=100\times \left(\mathrm{wet}\;\mathrm{weight}\;\mathrm{of}\;\mathrm{the}\;\mathrm{liver}/W_{f}\right);$$

$$\mathrm{IPR}\left(\%\right)=100\times \left(\mathrm{intraperitoneal}\;\mathrm{fat}\;\mathrm{weight}/W_{f}\right);$$

Where W_i_ is initial weight while W_f_ is final weight of fish (g) during the experiment; t is the duration of experiment (days); F is the weight of feed supplied to fish; and L is the average body length of fish (cm).

Nutrient Retention Efficiency (RE) was calculated as:

$$\mathrm{RE}\;\left(\%\right)=100\times \left(\left(W_{f}\times N_{f}\right)-\left(W_{i}\times N_{i}\right)\right)\times {\left(F\times N_{\mathrm{diet}}\right)}^{-1}$$

Where: N_diet_ is the nutritional content (protein and amino acid) of the diet which takes into account the apparent digestibility coefficient (ADC) of the nutrients (protein and amino acid), and N_i_ and N_f_ represent the initial and final concentration of the nutrients (protein and amino acid) in the whole minced fish.

### Biochemical analysis

Approximate composition of diets, whole-body of fish, dorsal muscle, and feces were tested by adopting the approach of Association of Official Analytical Chemists (AOAC) [29]. Dietary gross energy was tested using adiabatic bomb calorimeter (C2000, IKA Werke GmbH & Company, Staufen, Germany). The AA compositions of the diets, muscle, and feces were detected using an automatic AA analytical approach (Hitachi 835–50, Hitachi Co, Ltd, Tokyo, Japan). There are two types of ANFs in the soybean products. The first type is the antigen proteins such as glycinin, β-conglycinin, trypsin inhibitor, lectin, etc. The second type is the oligosaccharides such as oligosaccharides, stachyose, raffinose, etc. Glycinin, β-conglycinin, trypsin inhibitor, and lectins in the SPC and diets which were detected by indirect competition enzyme-linked immunosorbent assay (ELISA).Oligosaccharides, raffinose, stachyose, and sponins in SPC and diets were tested using High Performance Liquid Chromatography (HPLC). Urease activity was tested by the determination of ammonia production from urea hydrolysis by the enzyme.

The quantity of the chromium oxide in the diet and feces samples was analyzed using an atomic absorption spectrophotometer using the method of Williams and David [30]. The level of ADC was defined using the following formula:

$$\mathrm{ADC}\left(\%\right)=100\times \left(1-\left(\%\;{\mathrm{Cr}}_{2}O_{3}\;\mathrm{in}\;\mathrm{diet}/{\mathrm{Cr}}_{2}O_{3}\;\mathrm{in}\;\mathrm{feces}\right)\times \left(\%\;\mathrm{Nutrient}\;\mathrm{of}\;\mathrm{feces}/\mathrm{Nutrient}\;\mathrm{of}\;\mathrm{diet}\right)\right)$$

### Statistical analysis

Data was presented as the means ± standard deviation (SD, n = 3). After normality and heterogeneity of variance tests, one-way analysis of variance (ANOVA) was used for the comparison of the mean values (SPSS19.0, SPSS Inc., Chicago, USA). Statistically significant differences were described as *p* < 0.05. Correlation analysis was carried out between DFI and specific ANFs. The regression models and correlation analysis were established using Origin 9.0 (OriginLab Corporation, USA).

### Ethical statement

All applicable international, national, and/or institutional guidelines for the care and use of animals were followed by the authors. The use of animal was approved by the Institutional Animal Care and Use Committee of Hainan Tropical Ocean University.

## Results

### Growth performance and biometry

**Fig 1:**
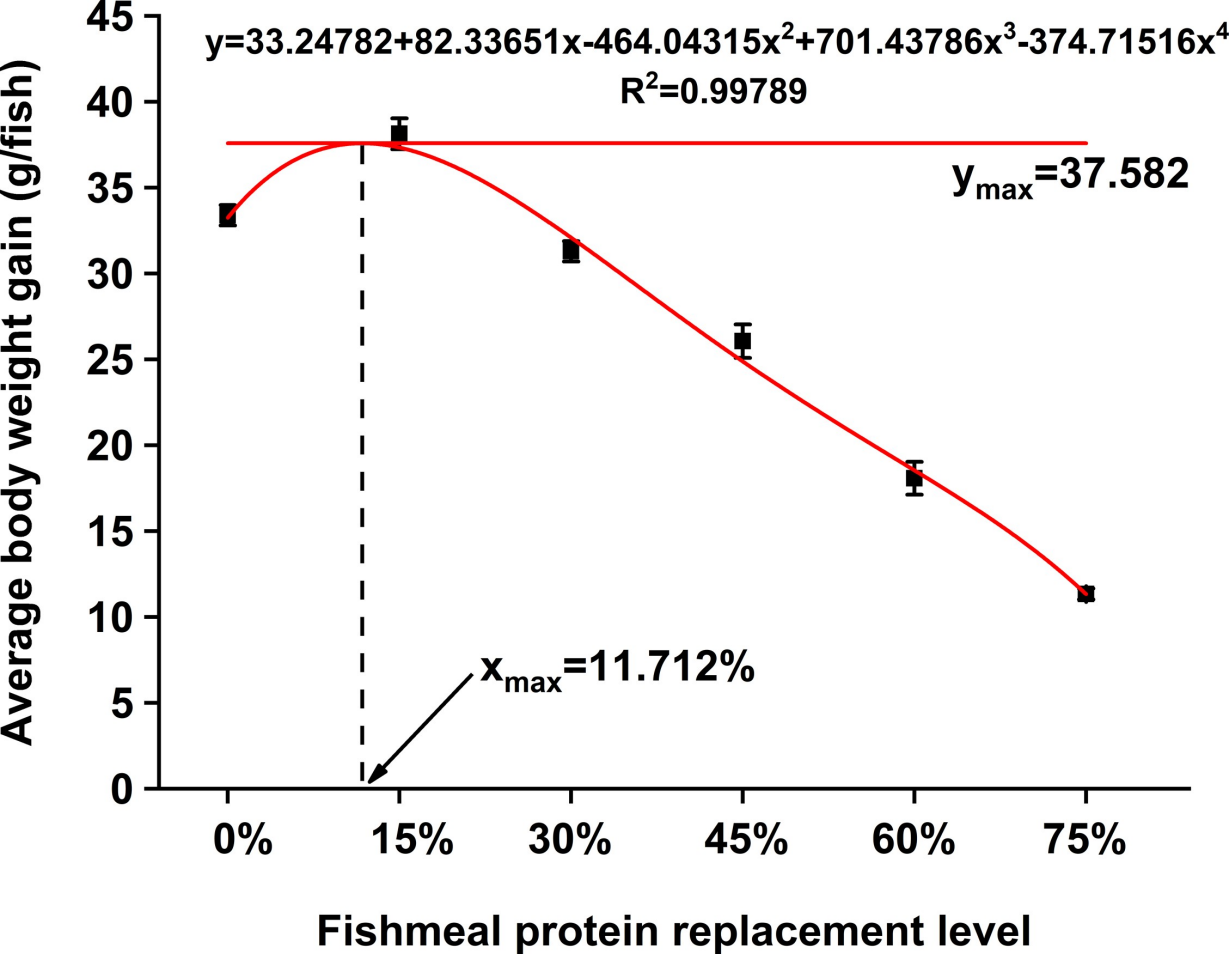
Quadratic regression model was established on average body weight gain (y-axis) in response to fishmeal protein replacement level (x-axis) by SPC.

**Fig 2:**
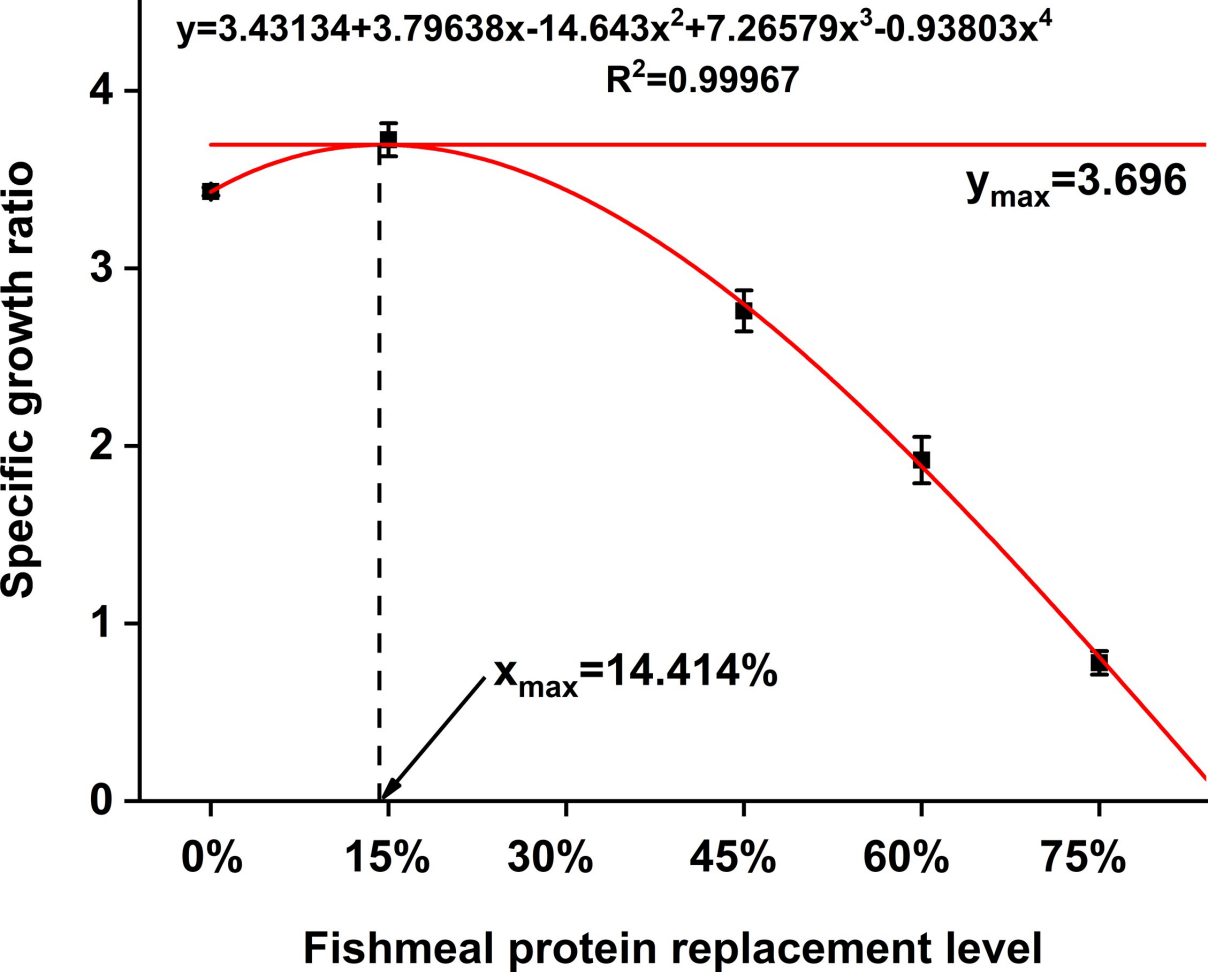
Quadratic regression model was established on specific growth ratio (y-axis) in response to fishmeal protein replaced (x-axis) by SPC.

Results of the growth performance are shown in Table 3. At the end of the trial, survival of the fish fed with different diets was significantly affected based on the concentration of dietary SPC (*P* < 0.05). The survival of juvenile pearl gentian grouper was higher in groups SPC 15, SPC 30, and SPC 45 (from 94% to 96%) as compared to SPC 60 and SPC 75 (*P* < 0.05). The BWG, WGR, SGR, and PER increased in fish fed with diet SPC 15, but gradually decreased with further increase of SPC concentration (*P < 0*.*05*). Conversely, the FCR values significantly decreased in groups SPC15, SPC 30, and SPC 45, but increased in the SPC 75 group (*P < 0*.*05*) which implied that SPC 15, SPC 30, and SPC 45 had a significantly higher feed conversion rate. The DFI was significantly higher in SPC 0 as compared to the groups treated with SPC (*P* < 0.05) implying that there was a significant effect of dietary SPC on DFI of the fish. Moreover, high SPC inclusion (SPC 75) clearly reduced the CF compared to the other treatments (*P<0*.*05*). HSI of fish was significantly higher in group SPC 0 as compared to SPC 30 and SPC 75 (*P<0*.*05*) but had no significant difference with SPC 15, SPC 45, and SPC 60. IPR fluctuated among the fish fed with different diets. According to the regression model of BWG (y-axis) corresponding to SPC replacement levels (x-axis) (Fig 1), optimal SPC replacement level was 11.71%. However, based on SGR (y-axis) and dietary SPC replacement levels (x-axis), it was determined to be 14.41% (Fig 2).

**Table 3:** Growth and biometry of the juvenile pearl gentian grouper fed with diets containing various concentrations of FM replaced with SPC.

Items	Diets					
	SPC0	SPC15	SPC 30	SPC45	SPC60	SPC75
W_i_ (g)	7.97 ± 0.00	7.98 ± 0.04	8.04 ± 0.03	8.01 ± 0.01	8.02 ± 0.01	8.01 ± 0.01
W_f_ (g)	41.29 ± 0.66^b^	46.12 ± 0.78^a^	39.34 ± 0.54^c^	34.23 ± 0.89^d^	26.14 ± 0.95^e^	19.53 ± 0.32^f^
BWG (g)	33.32 ± 0.60^b^	38.14 ± 0.90 ^a^	31.30 ± 0.60^c^	26.22 ± 0.98 ^d^	18.12 ± 0.95^e^	11.52 ± 0. 32^f^
WGR (%)	3.76 ± 0.13^b^	4.53 ± 0.33^a^	3.62 ± 0.12^b^	3.05 ± 0.29^c^	1.88 ± 0.17^d^	1.04 ± 0.06^e^
SGR (% d^−1^)	3.42 ± 0.02^b^	3.72 ± 0.09^a^	3.24 ± 0.06^c^	2.76 ± 0.12^d^	1.92 ± 0.13^e^	0.78 ± 0.07^f^
PER	1.69 ± 0.07^c^	2.32 ± 0.15^a^	2.21 ± 0.09^ab^	2.04 ± 0.17^b^	1.62 ± 0.14^c^	1.11 ± 0.07^d^
DFI (% d^−1^)	3.98 ± 0.11^a^	3.07 ± 0.13^bc^	2.95 ± 0.08^c^	3.07 ± 0.15^bc^	3.05 ± 0.12^bc^	3.09 ± 0.07^bc^
FCR	1.27 ± 0.05^b^	0.93 ± 0.06^a^	0.96 ± 0.04^a^	1.07 ± 0.09^a^	1.32 ± 0.12^b^	1.96 ± 0.13^c^
Survival %	91.11 ± 1.92^bc^	95.55 ± 3.85^ab^	94.44 ± 1.93^ab^	96.67 ± 3.34^a^	88.89 ± 1.93^cd^	85.56 ± 1.93^d^
Biometric indices						
CF (g cm^−3^)	1.73 ± 0.13^a^	1.65 ± 0.14^ab^	1.61 ± 0.19^ab^	1.61 ± 0.14^ab^	1.56 ± 0.15^b^	1.41 ± 0.20^c^
HSI (%)	2.97 ± 0.38^a^	2.47 ± 0.58^ab^	2.17 ± 0.56^b^	2.58 ± 0.54^ab^	2.46 ± 0.61^ab^	2.12 ± 0.61^b^
IPR (%)	1.41 ± 0.41^b^	1.68 ± 0.59^ab^	2.03 ± 0.58^a^	1.78 ± 0.33^ab^	1.69 ± 0.52^ab^	0.93 ± 0.57^c^

Values (mean ± SD, n = 3) within the same row with different letters are significantly different (*P* < 0.05). Absence of letters indicates no significant difference between treatments. W_i_: initial weight; W_f_: final weight of fish; BWG: average body weight gain; WGR: Weight gain ratio; SGR: specific growth rate, survival; PER: protein efficiency ratio; DFI: Daily feed intake; FCR: feed conversion ratio; SR: survival rate; CF: Condition factor; HIS: hepatosomatic index; IPR: intraperitoneal ratio.

### Nutritional composition

The approximate composition of the whole body and muscle are displayed in Table 4. In dorsal muscle of the fish, moisture and protein content were affected based on the concentration of dietary SPC (*P* < 0.05) while other compositions were not influenced. There was no significant difference in the crude protein of the dorsal muscle in the fish fed with SPC 0, SPC 15, and SPC 30 diet. However, those values were significantly higher as compared to fish fed with SPC 45, SPC 60, and SPC75 in which with the increasing dietary SPC concentration. The moisture of the dorsal muscle showed an opposite trend.

**Table 4:** Proximate composition of the muscle and the whole-body in juvenile pearl gentian grouper fed with diets containing various concentrations of FM replaced with SPC.

Items	Diets					
	SPC0	SPC15	SPC 30	SPC45	SPC60	SPC75
Proximate composition of muscle (% WW^(1)^)						
Moisture	77.23 ± 0.39^bc^	77.04 ± 1.09^c^	77.66 ± 0.92^bc^	78.10 ± 0.97^ab^	78.90 ± 0.84^a^	78.60 ± 0.75^a^
Protein	19.76 ± 0.53^a^	19.82 ± 0.96^a^	19.40 ± 0.80^a^	17.76 ± 0.59^b^	18.20 ± 0.74^b^	17.96 ± 0.64^b^
Lipid	1.99 ± 0.23	1.87 ± 0.88	2.06 ± 0.20	2.00 ± 0.11	1.89 ± 0.12	1.90 ± 0.19
Ash	1.36 ± 0.01	1.39 ± 0.02	1.36 ± 0.01	1.39 ± 0.02	1.34 ± 0.02	1.38 ± 0.02
Proximate composition of whole body (% WW)						
Moisture	69.73 ± 0.38^a^	69.74 ± 0.55^a^	70.36 ± 0.51^b^	70.67 ± 0.11^b^	70.64 ± 0.18^b^	71.88 ± 0.51^c^
Protein	18.01 ± 0.44^b^	18.05 ± 0.38^b^	19.17 ± 0.46^a^	18.69 ± 0.91^a^	17.21 ± 0.29^c^	16.95 ± 0.31^c^
lipid	5.17 ± 0.41^b^	5.58 ± 0.25^a^	5.49 ± 0.39^ab^	5.11 ± 0.38^b^	5.37 ± 0.17^ab^	5.44 ± 0.51^ab^
Ash	5.77 ± 0.09^a^	5.28 ± 0. 02^b^	4.87 ± 0.02^c^	4.66 ± 0.02^c^	4.43 ± 0.02^cd^	4.05 ± 0.00^d^

Values (mean ± SD, n = 3) within the same row with different letters are significantly different (*P* < 0.05). Absence of letters indicates no significant difference between treatments

^(1)^ WW is wet weight

In the whole body, the approximate components showed the significant differences (*P* < 0.05). The moisture content gradually increased with increasing SPC concentration and reached its maximum value in fish fed with SPC 75 diet. The fish fed with diet SPC 30—SPC 75 had significantly higher whole-body moisture than that in groups SPC 0 and SPC 15 (*P* < 0.05). The crude ash showed a contrary result. Crude protein content of the whole body in groups SPC 30 and SPC 45 was significantly higher (*P* < 0.05) among all the SPC treatments, and it decreased in groups SPC 60 and SPC 75.

AA composition of the muscle is presented in Table 5. Replacement of FM with SPC caused significant changes in the total AA composition of the fish muscle, including hydrolysis and free AA as compared to SPC 0 (*P* < 0.05). The histidine, lysine and methionine in the fish muscle decreased with increasing content of dietary SPC. As the first limited AA in SPC, the methionine in the fish muscle in group SPC 30 had significantly higher value than that in other groups. There was no significant difference in the value of methionine in groups SPC 0, SPC 15, SPC 45, and SPC 60, while methionine in SPC 75 had the lowest content (*P* < 0.05). Similarly, the lysine content in the fish muscle followed a trend similar to methionine but the highest lysine content was detected in SPC 15 treatment (*P* < 0.05). No significant differences were found in the levels of other AAs among the dietary treatments.

**Table 5:** Amino acid composition of muscle (g kg^−1^ dry weight for free amino acid and % for hydrolytic amino acid) of the juvenile pearl gentian grouper fed with diets containing various concentrations of FM replaced with SPC.

Items	Diets											
	SPC0		SPC15		SPC 30		SPC45		SPC60		SPC75	
	Hydrolytic	Free	Hydrolytic	Free	Hydrolytic	Free	Hydrolytic	Free	Hydrolytic	Free	Hydrolytic	Free
Polar basic amino acids												
Arginine	3.81±0.04^d^	0.77±0.01^a^	4.11±0.02^a^	0.31±0.01^c^	3.95±0.03^bc^	0.29±0.02^c^	4.05±0.06^ab^	0.77±0.01^a^	3.90±0.05^cd^	0.40±0.02^c^	3.98±0.05^bc^	0.65±0.02^b^
Histidine	1.33±0.01^a^	0.86±0.02^b^	1.26±0.03^ab^	0.99±0.03^a^	1.25±0.03^ab^	0.87±0.02^b^	1.24±0.04^b^	0.61±0.01^d^	1.23±0.00^b^	0.66±0.02^c^	1.09±0.07^c^	0.50±0.01^e^
Lysine	6.30±0.15^b^	4.93±0.10^a^	6.57±0.08^a^	4.40±0.02^b^	6.26±0.05^b^	3.68±0.01^c^	6.25±0.04^b^	3.32±0.02^d^	6.29±0.12^b^	3.10±0.01^e^	5.92±0.14^c^	1.22±0.02^f^
Polar acidic amino acids												
Aspartic acid	7.59±0.02^d^	0.66±0.01^a^	8.00±0.13^a^	0.62±0.01^b^	7.81±0.11^b^	0.37±0.01^c^	7.80±0.02^b^	0.33±0.02^c^	7.67±0.03^c^	0.32±0.02^c^	7.83±0.12^b^	0.33±003^c^
Glutamic acid	11.13±0.17^c^	5.22±0.10^b^	11.81±0.26^a^	5.42±0.13^a^	11.51±0.15^b^	4.17±0.12^c^	11.51±0.15^b^	3.97±0.12^d^	11.47±0.23^b^	3.10±0.20^f^	11.52±0.20^b^	3.73±0.13^e^
Non-polar amino acids												
Isoleucine	1.93±0.03^a^	1.83±0.01^a^	2.05±0.04^b^	1.67±0.04^b^	1.97±0.04^ab^	1.49±0.17^c^	1.95±0.05^ab^	1.17±0.21^d^	2.00±0.03^ab^	1.14±0.09^e^	1.99±0.05^ab^	1.15±0.15^de^
Leucine	5.48±0.23^b^	4.13±0.22^c^	5.89±0.22^a^	4.23±0.21^b^	5.63±0.19^b^	4.44±0.11^a^	5.64±0.18^b^	2.92±0.12^e^	5.57±0.24^b^	3.08±0.12^e^	5.47±0.12^b^	2.59±0.10^f^
Phenylalanin	2.71±0.02^ab^	2.16±0.17^a^	2.83 ±0.02^a^	2.11±0.12^a^	2.70±0.05^ab^	1.86±0.11^b^	2.74±0.02^ab^	1.42±0.10^d^	2.72±0.03^ab^	1.57±0.12^c^	2.69±0.05^b^	1.44±0.01^de^
Valine	2.21±0.01^ab^	2.86±0.01^a^	2.28±0.14^a^	2.63±0.02^b^	2.21±0.02^ab^	2.17±0.02^c^	2.17±0.05^b^	1.79±0.11^e^	2.27±0.02^a^	1.79±0.02^e^	2.25±0.03^ab^	2.03±0.10^d^
Glycine	3.50±0.21^c^	5.12±0.01^c^	3.71±0.03^b^	4.83±0.02^d^	3.98±0.10^a^	8.13±0.13^b^	4.06±0.09^a^	8.46±0.00^a^	3.69±0.17^b^	5.21±0.01^c^	3.73±0.13^b^	4.08±0.02^e^
Alanine	4.87±0.30^b^	4.88±0.01^a^	4.94±0.05^ab^	3.86±0.03^b^	4.91±0.26^ab^	3.73±0.13^b^	4.99±0.26^a^	3.78±0.13^b^	4.89±0.15^ab^	2.17±0.12^d^	4.93±0.03^ab^	3.18±0.12^c^
Polar neutral amino acids												
Methionine	1.95±0.03^b^	0.92±0.01^b^	1.94±0.03^b^	0.84±0.02^c^	2.06±0.04^a^	1.52±0.01^a^	1.94±0.02^b^	0.87±0.02^c^	1.98±0.02^b^	0.51±0.02^d^	1.75±0.03^c^	0.28±0.01^e^
Threonine	2.85±0.03^b^	0.78±0.01^c^	3.04±0.10^a^	0.64±0.05^d^	2.97±0.0^a^	1.19±0.05^b^	3.02±0.10^a^	1.66±0.02^a^	2.94±0.02^ab^	0.71±0.01^c^	2.94±0.05^ab^	0.42±0.01^e^
Serine	2.92±0.17^c^	0.51±0.01^b^	3.10±0.09^a^	0.29±0.02^d^	2.96±0.07^c^	0.35±0.01^c^	3.09±0.20^a^	0.88±0.02^a^	3.03±0.15^ab^	0.38±0.01^c^	2.99±0.06^ab^	0.27±0.01^d^
Tyrosine	2.13±0.12^b^	1.41±0.01^a^	2.23±0.25^a^	0.33±0.01^d^	2.11±0.19^b^	0.50±0.02^c^	2.10±0.21^b^	0.83±0.01^b^	2.10±0.17^b^	0.91±0.04^b^	2.16±0.32^b^	1.08±0.02^b^
Cysteine	0.51±0.03^b^	0.14±0.01	0.53±0.02^ab^	0.13±0.00	0.54±0.01^a^	0.13±0.01	0.52±0.01^ab^	0.13±0.01	0.52±0.05^ab^	0.14±0.00	0.51±0.02^b^	0.19±0.01
Taurine	0.58±0.02^a^	4.85±0.11^a^	0.39±0.01^c^	4.65±0.20^b^	0.42±0.01^c^	4.09±0.10^b^	0.40±0.06^c^	3.96±0.14^d^	0.51±0.02^b^	4.08±0.12^b^	0.49±0.02^b^	4.62±0.02^b^
ΣEAA	28.57±0.20^bc^	19.25±0.07^a^	29.97±0.16^a^	17.82±0.04^b^	28.99±0.17^b^	17.50±0.10^b^	29.00±0.21^b^	14.53±0.04^c^	28.57±0.09^bc^	12.96±0.03^d^	28.09±0.15^d^	10.28±0.04^e^
ΣNEAA	33.23±0.02^d^	22.79±0.05^a^	34.74±0.20^a^	20.13±0.12^c^	34.26±0.13^b^	21.46±0.13^b^	34.46±0.13^ab^	22.33±0.15^a^	33.89±0.05^c^	16.84±0.15^d^	34.16±0.14^bc^	17.47±0.17^d^
ΣAA	61.74±0.23^e^	42.04±0.11^a^	64.71±0.35^a^	37.95±0.02^c^	63.25±0.26^bc^	38.97±0.12^b^	63.46±0.33^a^	36.87±0.04^d^	62.76±0.11^cd^	29.80±0.05^e^	62.25±0.22^de^	27.75±0.13^f^
ΣDAA	27.09±0.03^e^	15.88±0.01^c^	28.50±0.13^a^	14.73±0.04^d^	28.22±0.14^bc^	16.39±0.03^b^	28.35±0.13^ab^	16.54±0.02^a^	27.72±0.02^d^	11.34±0.02^e^	28.02±0.10^c^	11.31±0.04^e^
ΣEAA/ΣAA	0.46±0.01	0.46±0.01^a^	0.46±0.01	0.47±0.02^a^	0.46±0.01	0.45±0.01^ab^	0.46±0.01	0.39±0.01^c^	0.46±0.01	0.43±0.01^ab^	0.45±0.01	0.37±0.02^c^
ΣEAA/ΣNEAA	0.86±0.02	0.84±0.01	0.86±0.01	0.88±0.02	0.85±0.01	0.82±0.01	0.84±0.02	0.65±0.02	0.85±0.02	0.77±0.02	0.82±0.01	0.59±0.02
ΣDAA/ΣAA	0.44±0.02	0.38±0.01	0.44±0.02	0.38±0.01	0.45±0.01	0.42±0.01	0.44±0.01	0.45±0.02	0.44±0.01	0.38±0.02	0.45±0.01	0.41±0.02

Values (mean ± SD, n = 3) within the same row with different letters are significantly different (*P* < 0.01). Absence of letters indicates no significant difference between treatments. ΣAA = total amino acid. ΣEAA = total essential amino acid. ΣNEAA = total nonessential amino acid. ΣDAA = delicious amino acid

Fish fed with SPC 15 diet had the significantly higher ΣEAA than other treatments in which ΣEAA had no significant difference, but with the increasing levels of SPC, the ΣEAA decreased, and lowest ΣEAA content was observed in fish fed with SPC 75 diet. Fish fed with SPC 15 and SPC 45 diet had the highest ΣAA and ΣNEAA (*P*<0.05). ΣEAA/ΣAA, ΣEAA/ΣNEAA, and ΣDAA/ΣAA in the muscle of the fish presented no significant differences among all the treatments.

Free lysine, aspartic acid, glutamic acid, isoleucine, leucine, valine, and alanine in the fish muscle significantly decreased with an increase in dietary SPC (*P*<0.05). Free methionine in the muscle of the fish fed with diet SPC 30, followed by that of fish fed with diet SPC 0 and fish fed with diet SPC75 hold the significant lower value (*P*<0.05). Free ΣEAA, ΣNEAA and ΣAA in fish fed with SPC 0 had a significantly higher value as compared to SPC dietary treatments (*P*<0.05). Fish fed with diet SPC 45 had the highest free ΣDAA in the muscle, followed by the fish fed with diet SPC 30 while the fish fed with diet SPC 75 had the lowest value (*P*<0.05).

### Protein and individual AA ADCs and retention in muscle

The ADC of protein and nine AAs in the diets are presented in Table 6. With an increase in the concentration of SPC, the ADCs of dietary protein and AA significantly declined (*P*<0.05). The ADC of the dietary protein was significantly higher in diets SPC 0 and SPC 15 as compared to the other diets; while that of SPC 30 and SPC 45 were significantly higher than that of SPC 60 and SPC 75 (*P*<0.05). The ADC of the essential amino acid (EAA) (lysine, arginine, isoleucine, leucine, valine, methionine, and threonine) and nonessential amino acid (NEAA) (aspartic acid, serine, alanine, and taurine) in diet SPC 0 was significantly higher as compared to other treatments, followed by SPC 15 and SPC 30, while SPC 75 had the lowest content (*P* < 0.05).

**Table 6:** Apparent digestibility of protein and amino acids (%) of the experimental diets containing various concentrations of FM replaced with SPC.

Items	Diets					
	SPC0	SPC15	SPC 30	SPC45	SPC60	SPC75
Protein	90.90 ± 0.12^a^	89.30 ± 0.21^a^	88.20 ± 1.01^b^	86.00 ± 2.02^b^	84.10 ± 0.98^c^	81.90 ± 0.84^c^
Polar basic amino acids						
Lysine	92.11 ± 0.46^a^	90.23 ± 1.12^b^	87.11 ± 0.82^c^	85.45 ± 0.54^d^	83.44 ± 0.30^e^	78.11 ± 0.76^f^
Arginine	90.23 ± 0.36^a^	89.05 ± 1.36^a^	87.11 ± 1.88^b^	85.45 ± 0.34^b^	83.23 ± 0.31^c^	81.95 ± 0.84^c^
Histidine	90.11 ± 0.37^a^	89.23 ± 0.57^ab^	87.75 ± 1.42^bc^	86.79 ± 0.57^c^	84.51 ± 0.31^d^	81.29 ± 1.37^e^
Polar acidic amino acid						
Aspartic acid	89.33 ± 0.99^a^	84.32 ± 0.34^b^	83.11 ± 0.71^b^	81.23 ± 0.37^c^	78.66 ± 0.29^d^	75.12 ± 1.48^e^
Glutamic acid	91.23 ± 0.47^a^	89.46 ± 0.45^b^	86.34 ± 0.89^c^	84.77 ± 1.43^d^	82.55 ± 0.31^e^	80.48 ± 0.79^d^
Non-polar amino acid						
Isoleucine	89.45 ± 0.42^a^	87.34 ± 0.91^b^	85.17 ± 0.42^c^	84.23 ± 0.35^cd^	82.98 ± 0.31^d^	79.98 ± 1.65^e^
Leucine	89.65 ± 0.41^a^	88.11 ± 0.66^ab^	86.99 ± 1.65^b^	85.27 ± 1.06^c^	83.45 ± 0.31^d^	81.45 ± 0.97^e^
Phenylalanine	88.79 ± 0.51^a^	86.29 ± 0.95^b^	84.52 ± 0.26^c^	82.34 ± 0.38^d^	80.27 ± 0.30^e^	79.21 ± 0.64^f^
Valine	89.17 ± 0.59^a^	87.65 ± 1.34^a^	85.34 ± 0.56^b^	83.11 ± 0.38^c^	81.65 ± 0.30^c^	78.12 ± 0.43^d^
Glycine	85.24 ± 1.03^a^	83.27 ± 0.38^b^	81.57 ± 1.22^c^	79.46 ± 1.35^d^	78.24 ± 0.29^d^	76.53 ± 0.85^e^
Alanine	87.79 ± 0.35^a^	85.76 ± 1.20^b^	82.99 ± 1.76^c^	81.65 ± 1.44^cd^	80.45 ± 0.30^d^	75.56 ± 0.44^e^
Polar neutral amino acid						
Methionine	93.24 ± 0.80^a^	90.56 ± 0.57^b^	87.16 ± 0.52^c^	83.44 ± 0.54^d^	81.21 ± 0.30^e^	78.55 ± 0.76^f^
Threonine	89.12 ± 0.80^a^	85.34 ± 0.45^b^	83.21 ± 0.65^c^	81.60 ± 0.38^d^	78.99 ± 0.29^e^	75.34 ± 0.65^f^
Serine	88.39 ± 0.34^a^	86.39 ± 0.06^b^	85.45 ± 1.13^b^	82.11 ± 1.90^c^	79.65 ± 0.29^d^	74.56 ± 1.07^e^
Tyrosine	90.34 ± 1.49^a^	88.77 ± 1.32^a^	85.47 ± 1.02^b^	83.78 ± 1.99^b^	80.83 ± 0.30^c^	77.68 ± 1.90^d^
Taurine	89.66 ± 0.36^a^	86.34 ± 1.56^b^	84.38 ± 0.22^c^	81.34 ± 1.09^d^	78.30 ± 0.29^e^	75.34 ± 1.69^f^
Cysteine	90.37 ± 0.36^a^	89.11 ± 1.67^ab^	87.35 ± 2.90^abc^	85.99 ± 1.70^bc^	84.32 ± 0.31^cd^	81.78 ± 2.20^d^

Values (mean ± SD, n = 3) within the same row with different letters are significantly different (*p* < 0.01). Absence of letters indicates no significant difference between treatments

The protein and the individual EAA retention of the muscle had a trend similar to the AA composition of the muscle (Table 7). Fish fed with SPC 15 (39.28%) and SPC 30 (37.92%) showed higher protein retention than other dietary treatments, followed by SPC 0; the lowest protein retention was in fish fed with SPC 75. The retention of EAA in the muscle of the fish fed with SPC 15 showed the highest value, followed by that of fish fed with SPC 30, whereas fish fed with SPC 75 diet had the lowest EAA retention (*P* < 0.05).

**Table 7:** Protein and individual essential amino acid (EAA) retention (%) in the muscle of the juvenile pearl gentian grouper fed with diets containing various concentrations of FM replaced with SPC.

Items	Diets					
	SPC0	SPC15	SPC 30	SPC45	SPC60	SPC75
Protein	28.89 ± 0.17^b^	37.92 ± 1.69^ab^	39.28 ± 0.87^a^	24.77 ± 1.26^c^	26.02 ± 1.00^c^	18.07 ± 0.46^d^
Polar basic amino acid						
Lysine	48.70 ± 0.58^d^	70.78 ± 2.61^a^	62.21 ± 2.31^b^	56.00 ± 0.61^c^	46.78 ± 0.32^d^	31.77 ± 0.76^e^
Arginine	30.85 ± 0.58^c^	41.15 ± 2.22^a^	36.72 ± 1.22^b^	32.05 ± 0.34^c^	24.54 ± 0.20^d^	17.27 ± 0.40^e^
Histidine	38.93 ± 0.58^c^	44.09 ± 2.22^a^	41.67 ± 1.12^b^	34.34 ± 1.10^d^	25.37 ± 0.14^e^	15.30 ± 0.68^f^
Non-polar amino acid						
Isoleucine	32.55 ± 1.47^cd^	46.40 ± 3.95^a^	40.40 ± 2.50^b^	34.53 ± 1.11^c^	29.04 ± 1.31^d^	22.99 ± 2.36^e^
Leucine	32.02 ± 1.21^d^	47.52 ± 3.52^a^	41.75 ± 1.27^b^	35.94 ± 0.59^c^	28.40 ± 0.60^e^	20.48 ± 0.76^f^
Phenylalanine	32.06 ± 0.72^c^	43.19 ± 3.06^a^	36.93 ± 2.31^b^	31.18 ± 0.45^c^	23.70 ± 0.48^d^	16.69 ± 0.70^e^
Valine	31.53 ± 0.40^c^	44.58 ± 2.49^a^	39.86 ± 1.58^b^	33.63 ± 1.06^c^	30.03 ± 0.76^c^	21.20 ± 0.28^d^
Polar neutral amino acid						
Methionine	43.45 ± 0.73^d^	75.09 ± 3.81^a^	64.41 ± 2.15^b^	51.76 ± 1.73^c^	50.08 ± 2.02^c^	37.30 ± 2.02^e^
Threonine	33.08 ± 0.28^d^	43.21 ± 1.75^a^	42.52 ± 0.92^a^	38.42 ± 0.81^c^	29.09 ± 0.80^e^	20.62 ± 1.51^f^

Values (mean ± SD, n = 3) within the same row with different letters are significantly different (*P* < 0.01). Absence of letters indicates no significant difference between treatment

## Discussion

It is known that fish can adapt to different nutritional conditions. In the past, plant proteins have been widely used in many fish diets for the partial or total replacement of FM, which may be one of the options to reduce the production costs in the aquaculture industry [31]. Among the plant proteins, soy products are nutritionally superior ingredients of feeds for aquatic animals [32]. Several studies have reported that when dietary SPC inclusion was below 60% a satisfactory growth and feed utilization was obtained in juvenile cobia [33] and juvenile starry flounder [19]; while further increase of SPC inclusion in the diet led to lower diet utilization and higher mortality in the fish. However, Zhao et al. (2010) showed that the survival and SGR of Nile tilapia was not affected even by the total replacement with SPC even the total replacement [34]. In the present study, fish fed the 15% SPC inclusion diet had a relatively better growth performance, and other treatments showed a gradual decrease with increasing SPC concentration.

According to the regression model, the FM replacement level by SPC was 11.7% and 14.4%. If taking this fish growth, diet utilization, and regression coefficient of fitting into account, the optimum FM replacement level by SPC was 30%. This implies that FM and SPC in diet for this fish were 18g 100g ^-1^and 45.5 g 100g ^-1^, respectively and the ratio of animal protein/vegetable protein (PA/PV) was 2.5:1.

Some fish, such as juvenile starry flounder and juvenile cobia can tolerate 40–75% SPC (FM 40.8 g 100g ^-1^ and SPC 27.92 g 100g ^-1^, FM16.0 g 100g ^-1^ and SPC 49.4g 100g ^-1^) and yellowtail kingfish (*Seriola lalandi*, *FM 35*.*5* g 100g ^-1^ and 36.5g 100g ^-1^) [19, 33, 35]. However, similar to our results, low tolerance of less than 30% SPC was reported in gilthead sea bream (*Sparus aurata* L., FM 47g 100g ^-1^ and SPC 20g100g ^-1^) [36], juvenile yellowtail kingfish (*Scophthalmus maximus*, FM 36 g 100g ^-1^ and solvent extracted soybean meal 10 g 100g ^-1^) [37], *Florida pompano (Trachinotus carolinus*, 24 g 100^-1^g) [38], and totoaba juveniles (*Totoaba macdonaldi*, *FM* 43 g 100g ^-1^ and 19.5g 100g ^-1^) [39], which revealed that the growth of these fish was inversely proportional to the level of dietary substitution of SPC and suggested that high SPC in the feed would reduce the growth of the fish, especially the carnivorous fish. Thus, FM is still the major protein sources for carnivorous.

Some reports argued that lower growth performance may be related to a decrease in feed intake rather than nutritional imbalance or deficiency [36]. This was suggested because as alternative feed, plant protein is usually less palatable than fishery products to fish [40]. In this present study, when fish were fed diets with high levels of replacement of FM with SPC, a reduction in DFI was observed which could cause reduced growth [36, 41]. Similar phenomenon was observed in juvenile starry flounder [32] and Japanese flounder [41], which were fed diets with over 50% FMP replaced with SPC. It is also worth noting that high SPC inclusion (above 60%) caused reduction in diet utilization, as reflected by an increase in FCR in our work. The reduced feed utilization further depressed the growth of pearl gentian grouper. A similar phenomenon was reported in juvenile starry flounder [19] and rainbow trout [42] that were fed diets with over 50% FM replaced with SPC.

The reduction in growth performance, dietary palatability, and feed utilization in SPC-rich diet could have resulted from the ANFs [43] and lower content of amino acids [8, 41]. In this study, the ANFs increased with the increasing replacement of FM by SPC, especially the phytic acid, trypsin inhibitor, raffinose, and glycinin. The effect of phytic acid and trypsin inhibitor on feed intake and growth depends primarily on the amount in the diet and on the presence or absence of a distinct stomach [44]. A correlation analysis of the feed intake and the phytic acid content of the experimental diets (SPC were used to replace 0%, 30%,60% and 100% FM; phytic acid were 4.9, 7.8, and 12.3g kg^-1^, respectively) indicated a negative correlation, r = −0.9 (n = 21) [36]. In this study, the correlation analysis of DFI, the phytic acid, trypsin inhibitor, raffinose, and glycinin also revealed a moderately negative correlation, r = −0.57, r = −0.41, r = −0.55, r = −0.55 (n = 6).

The content of phytic acid in SPC-rich diet in our work were notably lower than that reported in other studies for other fish which reduced the growth and feeding efficiency of fish, like the agastric common carp (*Cyprinus carpio*, 0.5 or 1%) [45], catfish (*Ictalurus punctatus*, 2.2%) [46], and juvenile chinook salmon (*Oncorhynchus tshawytscha*, 2.6% phytate)[47]. Storebakken et al. (1998) demonstrated that the inclusion of soy concentrates with high phytate concentration (18 g/kg) in the diets of Atlantic salmon (*Salmo salar*) led to a reduction in the bioavailability of Phosphorus, Calcium, Magnesium, and Zinc [15].

However, diets containing up to 1.5% phytic acid had no effect in catfish [46, 48]. Thus, inclusion of 0.8% phytic acid in the diet of Atlantic salmon was below the level that would depress its growth [44]. In this study, the content of trypsin inhibitor in SPC-rich diet was also lower than the tolerance level which had negative effects on the growth of other fish such as Atlantic salmon (*Salmo salar*, 5mg/g, trypsin inhibitor) [49] and channel catfish (3.2 mg/g trypsin inhibitor, 3.2mg/g) [50]. However, it needs further investigations to evaluate the separate effects of phytic acid or trypsin inhibitor and addition of plant meals containing phytic acid or trypsin inhibitor.

In this study, along with the growth performance of the fish, the crude protein in the muscle of the fish fed a diet with 45–75% SPC replacement were also significantly decreased. Also, the whole-body protein of the fish fed with a diet with 60%-75% SPC replacement had significantly lower values. Lack of methionine and lysine in the SPC-based diet might result in poor growth performance [41] and EAA imbalance in the fish [51]. This could be used to explain the relatively better growth performance of the fish fed with a diet containing 15% FM replacement by SPC, along with the higher content of lysine and methionine in SPC15 and SPC 30 fish muscle than others in the SPC-based diet. Free AA in the muscle of the fish may also connect with the imbalance in dietary AA. Consequently, the supplementation of methionine or lysine in diets which had high levels of SPC replacement was needed [34, 41, 52]. Thus, the addition of feed attractants and AA in low-FM-content aquaculture feeds may be useful for increasing their utilization [39, 34]. In the present study, both the SPC 0–75 diets were without crystalline AA supplementation. Further experiments need to be conducted on the replacement of FM with SPC along with the supplementation of crystalline AA.

It has been reported that no effects of dietary SPC were observed on protein and AA digestibility [35, 53]. In contrast, in this study the ADC of protein and AA declined with the increasing replacement level of SPC, which was similar to the results of the studies in rainbow trout [42], Atlantic salmon [54], and Japanese flounder [41]. It is hypothesized that unbalanced AA and ANFs in the high level of SPC-based diet may have a negative effect on the digestibility of crude protein and certain AA_S_ for fast-growing fish [55]. In this study, we also found that the retention of lysine, arginine, histidine, isoleucine, leucine, phenylalanine, valine, methionine, and threonine in the fish muscle were significantly influenced by the SPC treatments. This may be attributed to lysine, histidine, isoleucine, valine, glycine, alanine, and methionine imbalance in the diets which have high amounts of single plant proteins, which may be unfavorable for fish growth, muscle protein, and AA retention. Besides, carbohydrate removal from SPC-rich diet might be responsible for their ADC of protein and AA. On the contrary, a mixed protein source (corn gluten, wheat gluten, soybean meal, and rapeseed meal) could replace almost total FM (95%) in the diet of European sea bass [56]. Kissil and Lupatsch [57] also revealed that a mixture of plant protein concentrate (corn gluten, wheat gluten, soy protein concentrate) could substitute FM at 75–100% for gilthead sea bream.

In summary, juvenile pearl gentian grouper showed poor SPC tolerance. The maximum level of SPC substitution for FM in the fish diet, according to WG and SGR, was estimated to be 11–14%. However, a 30% SPC replacement revealed a positive influence on protein and AA retention. Thus, it suggested that the proportion of SPC instead of FM for juvenile pearl gentian grouper should be less than 30%. This work would provide a reference for use of SPC in pearl gentian grouper.
